# Roles of the low density lipoprotein receptor and related receptors in inhibition of lipoprotein(a) internalization by proprotein convertase subtilisin/kexin type 9

**DOI:** 10.1371/journal.pone.0180869

**Published:** 2017-07-27

**Authors:** Rocco Romagnuolo, Corey A. Scipione, Santica M. Marcovina, Matthew Gemin, Nabil G. Seidah, Michael B. Boffa, Marlys L. Koschinsky

**Affiliations:** 1 Department of Chemistry & Biochemistry, University of Windsor, Windsor, Ontario, Canada; 2 Department of Medicine, Northwest Lipid Research Laboratories, University of Washington, Seattle, Washington, United States of America; 3 Laboratory of Biochemical Neuroendocrinology, Institut de Recherches Cliniques de Montréal (IRCM), Montréal, Québec, Canada; 4 Department of Biochemistry, Schulich School of Medicine & Dentistry, University of Western Ontario, London, Ontario, Canada; 5 Robarts Research Institute and Department of Physiology & Pharmacology, Schulich School of Medicine & Dentistry, University of Western Ontario, London, Ontario, Canada; Centro Cardiologico Monzino, ITALY

## Abstract

Elevated plasma concentrations of lipoprotein(a) (Lp(a)) are a causal risk factor for cardiovascular disease. The mechanisms underlying Lp(a) clearance from plasma remain unclear, which is an obvious barrier to the development of therapies to specifically lower levels of this lipoprotein. Recently, it has been documented that monoclonal antibody inhibitors of proprotein convertase subtilisin/kexin type 9 (PCSK9) can lower plasma Lp(a) levels by 30%. Since PCSK9 acts primarily through the low density lipoprotein receptor (LDLR), this result is in conflict with the prevailing view that the LDLR does not participate in Lp(a) clearance. To support our recent findings in HepG2 cells that the LDLR can act as a *bona fide* receptor for Lp(a) whose effects are sensitive to PCSK9, we undertook a series of Lp(a) internalization experiments using different hepatic cells, with different variants of PCSK9, and with different members of the LDLR family. We found that PCSK9 decreased Lp(a) and/or apo(a) internalization by Huh7 human hepatoma cells and by primary mouse and human hepatocytes. Overexpression of human LDLR appeared to enhance apo(a)/Lp(a) internalization in both types of primary cells. Importantly, internalization of Lp(a) by LDLR-deficient mouse hepatocytes was not affected by PCSK9, but the effect of PCSK9 was restored upon overexpression of human LDLR. In HepG2 cells, Lp(a) internalization was decreased by gain-of-function mutants of PCSK9 more than by wild-type PCSK9, and a loss-of function variant had a reduced ability to influence Lp(a) internalization. Apo(a) internalization by HepG2 cells was not affected by apo(a) isoform size. Finally, we showed that very low density lipoprotein receptor (VLDLR), LDR-related protein (LRP)-8, and LRP-1 do not play a role in Lp(a) internalization or the effect of PCSK9 on Lp(a) internalization. Our findings are consistent with the idea that PCSK9 inhibits Lp(a) clearance through the LDLR, but do not exclude other effects of PCSK9 such as on Lp(a) biosynthesis.

## Introduction

Genetic studies performed within the last decade have provided conclusive evidence that elevated plasma lipoprotein(a) [Lp(a)] concentrations are a causal risk factor for coronary heart disease [1, 2]. Lp(a) contains a lipoprotein moiety indistinguishable from low density lipoprotein (LDL), but also contains the unique glycoprotein apolipoprotein(a) (apo(a)) [3]. Apo(a) is characterized by the presence of multiple copies of plasminogen-like kringle IV (KIV) sequences, followed by domains closely resembling plasminogen kringle V (KV) and an inactive protease domain [4]. The KIV domain can be sub-divided into ten types (KIV_1_-KIV_10_) based on differences in amino acid sequence [5]. Apo(a) can contain from as few as 3 to greater than 40 identically repeated KIV_2_ domains which accounts for the phenomenon of Lp(a) isoform size heterogeneity, a hallmark of this lipoprotein [6, 7]. A general inverse relationship between apo(a) size and Lp(a) plasma concentration has been well-established, with Lp(a) levels varying widely in the population [8]. It has been reported that the inverse correlation between apo(a) isoform size and plasma Lp(a) levels is primarily dictated by the level of production rather than catabolism of the particle [9, 10]. Up to 90% of the variation in Lp(a) levels is genetically determined based on variation in the apo(a) gene including its size heterogeneity [11]; this has presented challenges in the development of therapeutic strategies to lower Lp(a) [12].

The process of Lp(a) catabolism remains largely unclear. Various receptors have been proposed to mediate Lp(a) uptake by the liver including the LDL receptor (LDLR) [13–17], very low-density lipoprotein receptor (VLDLR) [18], [17, 19], megalin/gp330 [20], scavenger receptor class B type 1 [21], and plasminogen receptors [13]. Unlike LDL, the LDLR is not the major clearance receptor for Lp(a), although the exact role of the LDLR in Lp(a) catabolism remains a point of controversy. Cain and coworkers reported that while plasma clearance of Lp(a) in mice occurs primarily through the liver and is mediated by apo(a), the catabolism of Lp(a) in *Ldlr*^-/-^ mice was similar to that in wild-type mice, suggesting no role for the LDLR [22]. Similar results were observed in catabolic studies of Lp(a) in human subjects with FH [23]. Additionally, plasma Lp(a) concentrations are largely insensitive to statins [24]; as these drugs act by increasing the abundance of hepatic LDLR, this result again suggests to no role for the LDLR in Lp(a) catabolism. However, evidence in support of a role for the LDLR has also been reported. For example, Hofmann *et al*. found that Lp(a) clearance was significantly increased in mice overexpressing LDLR [25]. Additionally, several other *in vitro* and *in vivo* studies have shown that the LDLR can mediate Lp(a) binding and uptake [13–17]. Results of a cross-sectional analysis of 1,960 patients with familial hypercholesterolemia revealed that Lp(a) levels were significantly higher in patients with a null LDLR allele compared to control subjects [26], a finding that is in agreement with previous work [27].

Most recently, a number of studies have shown that Lp(a) levels in plasma can be reduced up to 30% using a proprotein convertase subtilisin/kexin type 9 (PCSK9) inhibitory monoclonal antibody [28–34]. PCSK9 acts as an endogenous regulator of LDLR levels and has been implicated in some cases of FH due to the dominant gain-of-function (GOF) mutations identified in the population [35]. GOF mutations increase the affinity of PCSK9 for the LDLR which results in a more rapid degradation of the LDLR and thus higher plasma LDL [35]. Conversely, loss-of-function (LOF) mutations in PCSK9 result in dramatically lowered plasma LDL [35]. [36]. Studies from our group and others, aimed at establishing a mechanism for how PCSK9 inhibitors lower plasma Lp(a) levels, found that Lp(a) uptake by the LDLR in cultured human hepatoma cells can be modulated by PCSK9 [34, 37]; this provides a mechanistic basis for the ability of PCSK9 inhibitors to lower Lp(a) levels in humans. It is possible that the LDLR is not a major clearance receptor for Lp(a) in normal circumstances, but becomes so in the setting of PCSK9 inhibitors coupled with optimal statin therapy, where competing levels of LDL in plasma would be minimized while hepatic LDLR abundance would be maximized [37]. In patients treated with a PCSK9 monoclonal antibody, the extent of Lp(a) lowering correlated with the extent of LDL lowering in some studies [30, 31, 34] but not others [33]. The basis for these conflicting results is not clear. While plasma LDL is predominately cleared through the LDLR [38, 39], it is not clear whether Lp(a) catabolism is also modulated by other receptors which are sensitive to PCSK9 degradation.

In the current study, we used both established human cell lines and primary human and mouse hepatocytes to further our understanding of how PCSK9 inhibitory antibodies lower plasma Lp(a) concentrations, in the context of the ongoing controversy surrounding the role of the LDLR in Lp(a) catabolism.

## Materials and methods

### Cell culture–established lines

Human embryonic kidney (HEK293) cells were maintained in MEM (GIBCO) containing 5% fetal bovine serum (FBS; GIBCO) and 1% antibiotic-antimycotic (GIBCO). Human hepatocellular carcinoma (Huh7 and HepG2) cells were obtained from the Japanese Collection of Research Bioresources and American Type Culture Collection (ATCC), respectively. Hepatocytes were maintained in MEM supplemented with 10% FBS (ATCC) and 1% antibiotic-antimycotic (GIBCO). CHO-K1 (normal) and CHO 13-5-1 (LRP-1-deficient) cells (a generous gift from David J. Fitzgerald, laboratory of Molecular Biology, NIH, USA) [40] were grown in DMEM/F12-Ham medium supplemented with 10% fetal bovine serum.

### Cell culture–primary cells

Primary human hepatocytes, from a male donor and of a unique lot number, were obtained from BioreclamationIVT and were cultured in InVitroGRO HT medium as per manufacturer’s instructions.

Mice in which both alleles of the *Ldlr* gene were inactivated (*Ldlr*^*tm1Her*^; hereafter referred to as *Ldlr*^-/-^) were obtained from Jackson Laboratories, as were wild-type mice of the background strain (C57BL/6). Primary hepatocytes from wild-type or *Ldlr*^-/-^ mice were prepared by a modified collagenase perfusion method [41]. Mice were subjected to deep anesthesia (pentobarbitol sodium diluted in saline at a dose of 50mg/kg); mice were determined to have met surgical plane anesthesia when toe pinch reflex was absent. Following thoracotomy and catheterization of the hepatic portal vein, the livers were perfused with 50 mL of HBS containing 0.5 mM EGTA at a flow rate of 8 mL/min, followed by 50 mL of HBS containing 5 mM CaCl_2_ and collagenase (from *Clostridium histolyticum*, 0.5 mg/mL) at a rate of 6 mL/min. After isolation, viability of hepatocytes was assessed by trypan blue exclusion staining (viability of >80% was considered acceptable) and the cells were plated at a density of 2.0 × 10^5^ cells/well into gelatin-coated 24-well plates in Williams E media (GIBCO) supplemented with 10% FBS (ATCC), 2 mM L-glutamine (GIBCO), and 1% antibiotic-antimycotic (GIBCO). Approximately two hours after seeding, non-adherent cells were aspirated from the plate and fresh medium was added. Transient transfections (see below) were conducted 1 hour later. The animal experiments described above were specifically approved by the University of Windsor Animal Use and Care Committee (protocol #14–24) and mice were maintained following the Canadian Council on Animal Care guidelines at the University of Windsor.

### Transient transfections

HepG2 cells, Huh7 cells, primary human hepatocytes, or primary mouse hepatocytes were transiently transfected using MegaTran 1.0 (Origene) as previously described [37]. Briefly, cells were seeded at a density of 2 × 10^5^ cells/well in a 6-well dish and transfected 24 hours later (with the exception being primary mouse hepatocytes as noted above) using 1.2 μg/well of plasmid with a 3:1 ratio of reagent to DNA. In all cases, cells were assayed 72 hours post transfection, with the exception of the primary mouse hepatocytes, which were assayed after 36 hours. Plasmids employed, each of which encoded full-length receptors, were pIR-LDLR-v5 or the parental vector pIR-v5 [40]; pCMV6-LRP-1 (RC218369); pCMV6-LRP-8 (RC220963); pCMV6-VLDLR (RC211796); or the parental vector pCMV6. All pCMV6-based plasmids were purchased from OriGene.

### Construction, expression, and purification of recombinant apo(a)

The construction of expression plasmids encoding recombinant apo(a) (r-apo(a)) variants (12K, 17K, 23K, and 30K), the establishment of cell lines stably expressing these variants, and the purification of r-apo(a) from conditioned medium harvested from these lines has been previously described [42]. In brief, the conditioned medium was subjected to lysine-Sepharose affinity chromatography and r-apo(a) was eluted using the lysine analogue ε-aminocaproic acid (ε-ACA). Following concentration and dialysis, protein concentrations were determined spectrophotometrically using pre-determined molar extinction coefficients [42]. The purity of r-apo(a) was assessed using SDS-PAGE followed by silver staining.

### Construction, expression, and purification of recombinant PCSK9

The construction of PCSK9, PCSK9 D374Y, PCSK9 L455X and PCSK9Δ33–58 expression plasmids in pIRES2-EGFP (Clontech) have been previously-described [43]. For recombinant expression, the PCSK9 variant cDNAs were inserted into pcDNA4C (Invitrogen) as previously described [36], such that the expressed proteins would contain a carboxyl-terminal 6× His tag. HEK293 cell lines stably expressing wild-type PCSK9 were constructed and recombinant PCSK9 variants were purified by affinity chromatography over Ni^2+^-Sepharose as previously described [37]. Concentrations were determined through bicinchoninic acid assay (BCA assay; Pierce) using BSA as a standard. PCSK9 proteins were stored in aliquots at -70°C until use, and purity was assessed by SDS-PAGE followed and silver staining.

### Purification of Lp(a)

For Lp(a) purification, blood was collected from a single healthy human volunteer (with written informed consent) with high levels of Lp(a) corresponding to a single 16-KIV isoform. Blood was drawn into BD Vacutainers containing sodium polyanethol sulfonate and acid citrate dextrose. Lp(a) was purified by NaBr-adjusted density flotation followed by size exclusion chromatography, as previously described [37]. Concentrations of Lp(a) were determined by BCA assay using BSA as a standard.

### Internalization assays

HepG2 cells, HUH7 cells, CHO cells, primary human hepatocytes, or primary mouse hepatocytes were seeded at a density of 2 × 10^5^ cells/well in a 24-well plate (precoated with 1 mg/mL gelatin or collagen in the case of primary human hepatocytes), in medium containing 10% lipoprotein-deficient serum (LPDS; prepared as previously described [13]) for 16 h. Cells were washed twice with OptiMEM (GIBCO) and treated with Lp(a) purified from human plasma (5–10 μg/mL) or r-apo(a) variants (100–200 nM) in the presence of 0, 1, 10, or 20 μg/mL purified recombinant PCSK9 in OptiMEM. After 4 h, cells were then sequentially washed as we have previously described [37] with PBS supplemented with BSA, PBS containing BSA and ε-ACA, acetic acid containing NaCl, and finally PBS. The cells were then lysed with lysis buffer (50 mM Tris pH 8.0 containing 1% NP-40, 0.5% sodium deoxycholate, 150 mM NaCl, 1 mM EDTA, 0.1% SDS, 1 mM PMSF, and 150 μg/mL benzamidine).

### Western blotting

Lysates were subjected to western blot analysis as previously described [37], using either mouse-anti human apo(a) a5 antibody (1:5,000 dilution) or mouse-anti human β-actin (GE Healthcare NA931; 1:15,000). After incubation with a horseradish peroxidase-linked goat anti-mouse IgG secondary antibody, immunoreactive bands were visualized using SuperSignal West Femto Maximum Sensitivity Substrate (Thermo Scientific) and the relative amounts of protein were determined by densitometric quantification on a FluorChem Q imager running Alpha View software (Alpha Innotech) or a Chemi-Doc MP imager running Image Lab software (Bio-Rad). Under these conditions, the densitometric signal is a linear function of the amount of immunoreactive material, with no effects of saturation apparent (S1 Fig). Protein internalization is represented as apo(a) signal relative to that of β-actin from the corresponding lysate sample, expressed as a fraction of apo(a) internalized in the untreated control. For experiments using HuH7 cell lines where PCSK9 levels were manipulated, or where hepatocytes were transfected with expression plasmids encoding lipoprotein receptors, blots were blocked for 1 hour in 5% milk in Tris-buffered saline with 0.05% Tween-20 then subjected to overnight incubation with either rabbit monoclonal anti-PCSK9 (Abcam ab181142; 1:2,000); mouse monoclonal anti-v5 (Thermo R96025; 1:2000); rabbit anti-LRP-8 (SantaCruz Biotechnology sc-20746, 1:250); rabbit anti-LRP1 (Abcam ab92544; 1:5,000); or mouse anti-vLDLR (SantaCruz Biotechnology sc-18824, 1;250). Blots were imaged as described above after incubation with the appropriate HRP-conjugated anti-mouse or anti-rabbit secondary antibody.

### Real-time qRT-PCR

Hepatocyte lysates were collected, after transfection and apo(a)/Lp(a) treatment, using RLT Plus buffer (Qiagen) supplemented with 1% (v/v) 2-mercaptoethanol. Total RNA was isolated from the lysates using the RNeasy Plus kit (Qiagen) as per the manufacturer’s protocol. LRP-8, LRP-1 or VLDLR mRNA expression was assessed by qRT-PCR utilizing primer pairs A/B, C/D, E/F, respectively (Table 1) and normalized to GAPDH using primer pair G/H (Table 1). All DNA primers utilized in this study were synthesized by Integrated DNA Technologies, Inc. Reactions were assembled in triplicate using the iTaq Universal SYBR Green One-Step kit (Bio-Rad) according to the manufacturer’s protocol, and performed on a Bio-Rad CFX Connect. Relative quantification of the respective mRNA was determined by normalization of the threshold cycle for each sample with that of GAPDH (primer set G/H; Table 1) from the same reaction well using Bio-Rad CFX Manager software version 3.1 (Bio-Rad). All mRNA expression data are reported as a fold-increase compared with the control sample from cells that were transfected with the pCMV6 parental plasmid.

**Table 1:** Oligonucleotide primers used in this study.

Primer	Sequence
**A**	5’- GCTACCCTGGCTACGAGATG-3’
**B**	5’- GATTAGGGATGGGCTCTTGC-3’
**C**	5’- TTTAACAGCACCGAGTACCAG-3’
**D**	5’- CAGGCAGATGTCAGAGCAG-3’
**E**	5’- CGCCTCTATTGGCTTGATTCTAA-3’
**F**	5’- TCCTACGATCTTGGCCATTCA-3’
**G**	5’- AGCCACATCGCTCAGACAC-3’
**H**	5’- GCCCAATACGACCAAATCC-3’

### Statistical methods

Western blot data were normalized to control conditions to allow data across multiple independent experiments to be compared. Significant differences compared to (normalized to 100%) were performed using the one-sample t-test, while pairwise comparisons not involving control conditions were performed using two-tail Student's t-test assuming equal variances. Multiple comparisons were performed using one-way ANOVA Tukey’s post hoc test All analyses were implemented on GraphPad Prism 7.00 software. Statistical significance was assumed at p < 0.05.

## Results

### Internalization of apo(a) and Lp(a) by Huh7 cells

**Fig 1:**
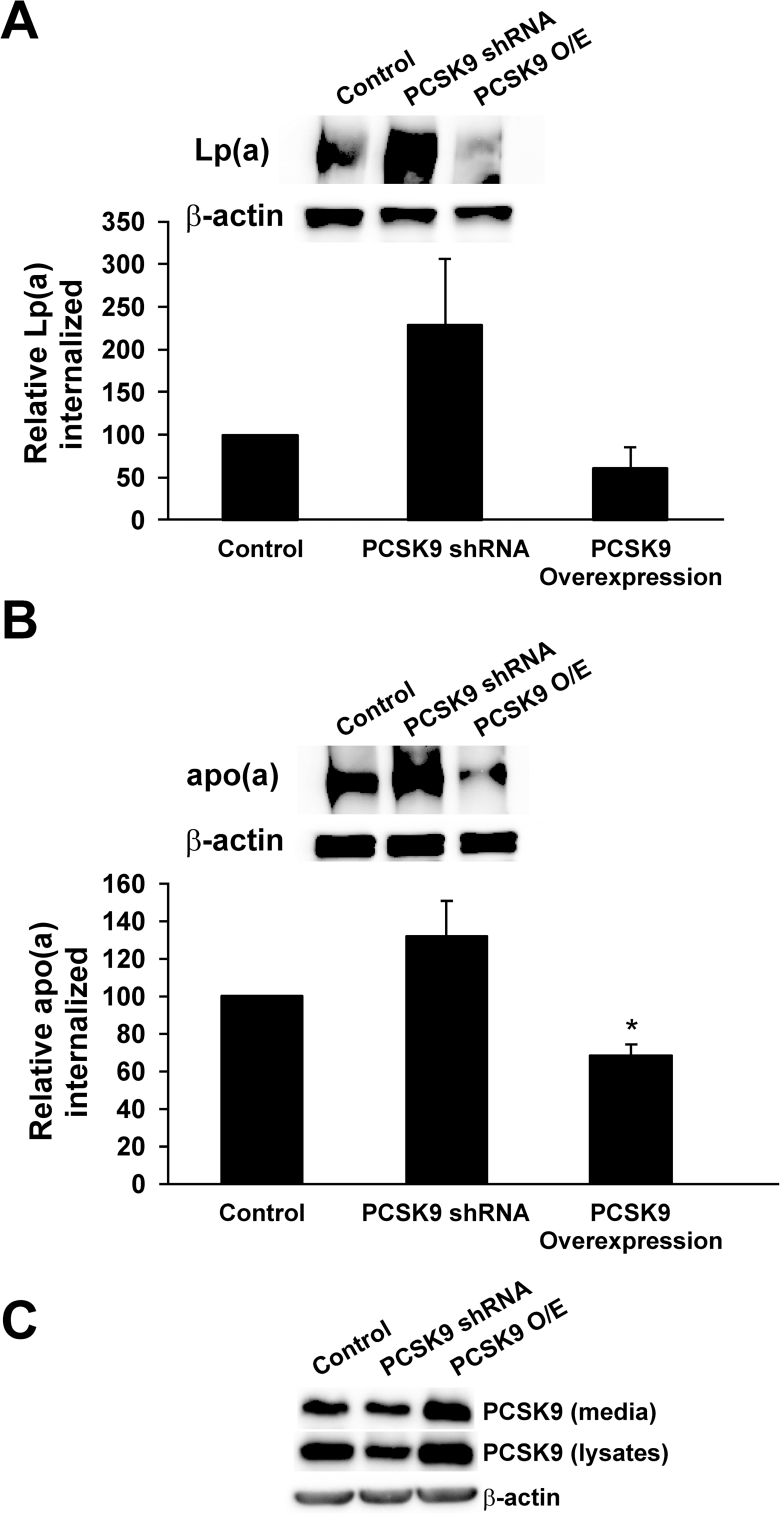
Internalization of Lp(a) by the Huh7 human hepatoma cell line. Huh7 cells were stably transfected with expression plasmids encoding either PCSK9 or shRNA against PCSK9, or were mock-transfected. Cells were incubated with 10 μg/mL human Lp(a) (A) or 200 nM 17K apo(a) (B) for 4 hours. Cells were extensively washed to remove any bound Lp(a) and lysed to determine the relative amount of internalized Lp(a) compared to β-actin using western blot analysis. Representative blots are shown. The results represent the means ± s.e.m. of at least 3 independent experiments. *: p < 0.05 for indicated pairwise comparisons by one-sample t-test.

Our previous studies [37] demonstrated that PCSK9 modulates apo(a) and Lp(a) internalization into HepG2 human hepatocellular carcinoma cells via the LDLR. To provide further evidence for this mechanism, we performed similar internalization assays in different hepatic cell types. We first utilized Huh7 cells, another human hepatoma cell line that has been used in studies of LDL catabolism [44, 45]. We used a similar protocol to our previous studies, in which purified 17K apo(a) or Lp(a) was incubated with the cells, which were then extensively washed to remove surface-associated proteins prior to lysis and measurement of apo(a)/Lp(a) internalization by western blot analysis. We manipulated PCSK9 activity either by transfecting the cells with an expression plasmid for PCSK9 or PCSK9 shRNA. We found that overexpression of PCSK9 appeared to reduce Lp(a) and apo(a) internalization, although only the latter effect reached significance (Fig 1). Knockdown of PCSK9 expression similarly appeared to increase Lp(a) and apo(a) internalization, although these did not reach statistical significance (Fig 1).

### Internalization of apo(a) by primary human hepatocytes

**Fig 2:**
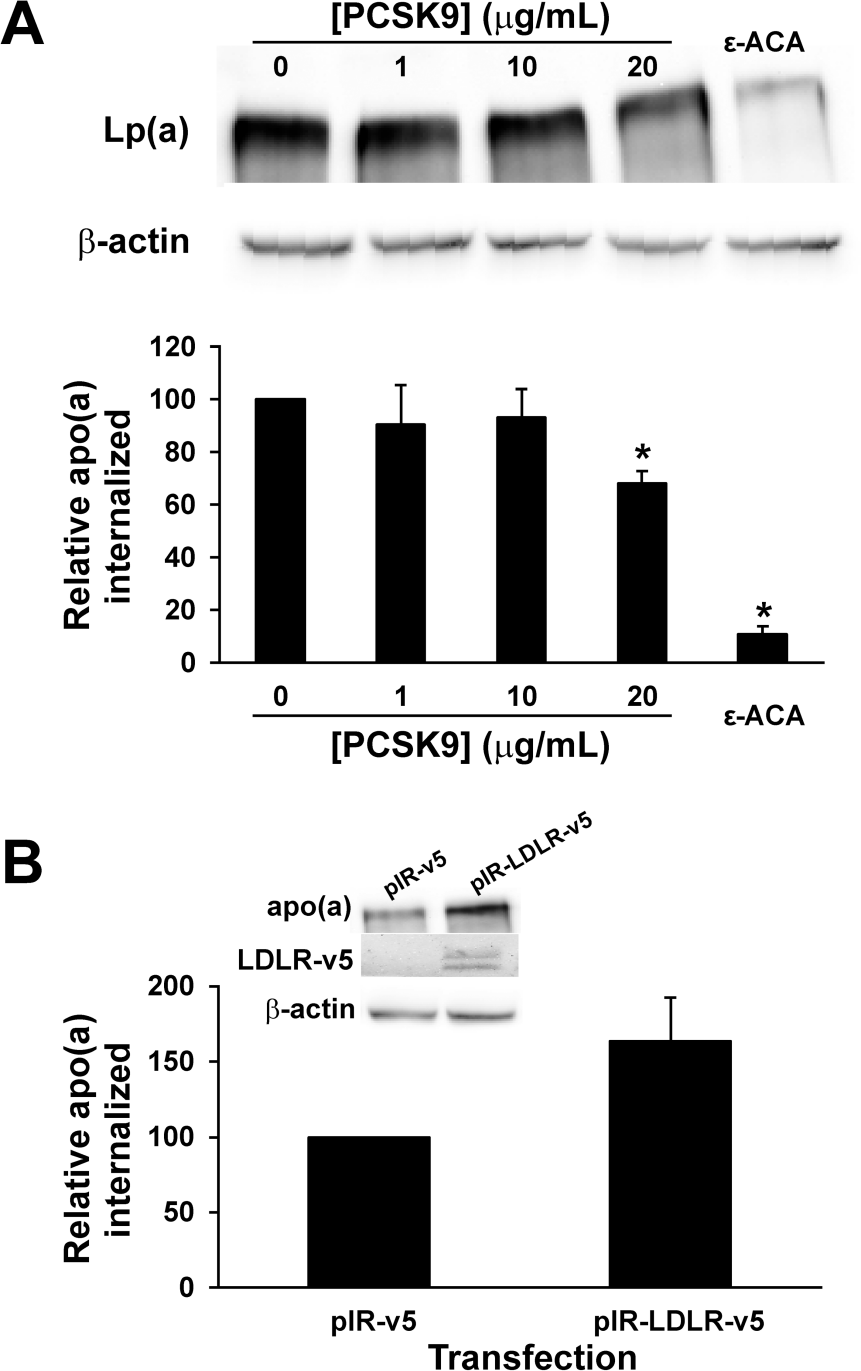
Internalization of apo(a) by primary human hepatocytes. (A) Hepatocytes were plated on a collagen matrix and then incubated with 200 nM 17K apo(a) in the absence or presence of the indicated concentrations of PCSK9 or 200 mM ε-ACA for 4 hours. Cells were extensively washed to remove any bound apo(a) and lysed to determine the relative amount of internalized apo(a) compared to β-actin using western blot analysis. Representative blots are also shown. The results represent the means ± s.e.m. of at least 3 independent experiments. *: p < 0.05 for indicated pairwise comparisons by one-sample t-test. (B) Hepatocytes were transiently transfected with an expression vector encoding human LDLR (pIR-LDLR-v5) or the corresponding empty expression vector. Internalization of 17K apo(a) was determined as described for Panel A. Also shown are representative blots probed with an anti-apo(a) antibody for apo(a) internalization and an anti-v5 antibody for LDLR overexpression.

We next attempted to use primary human hepatocytes to study the role of PCSK9 in apo(a)/Lp(a) internalization. We were only able to evaluate apo(a) internalization because we noticed an exceptionally high degree of non-specific association of Lp(a) with the cells (S2 Fig), which most likely reflects association with the extracellular matrix that must be added to the surface of the culture plates when seeding these cells (S3 Fig). We found that purified PCSK9, at the highest concentration tested, significantly decreased apo(a) internalization by these cells (Fig 2A). As we have found previously, addition of the lysine analog ε-ACA greatly diminished apo(a) internalization (Fig 2A). Moreover, overexpression of the LDLR in the primary human hepatocytes appeared to promote apo(a) internalization, although at borderline significance (p = 0.10) (Fig 2B).

### Internalization of Lp(a) by primary mouse hepatocytes

**Fig 3:**
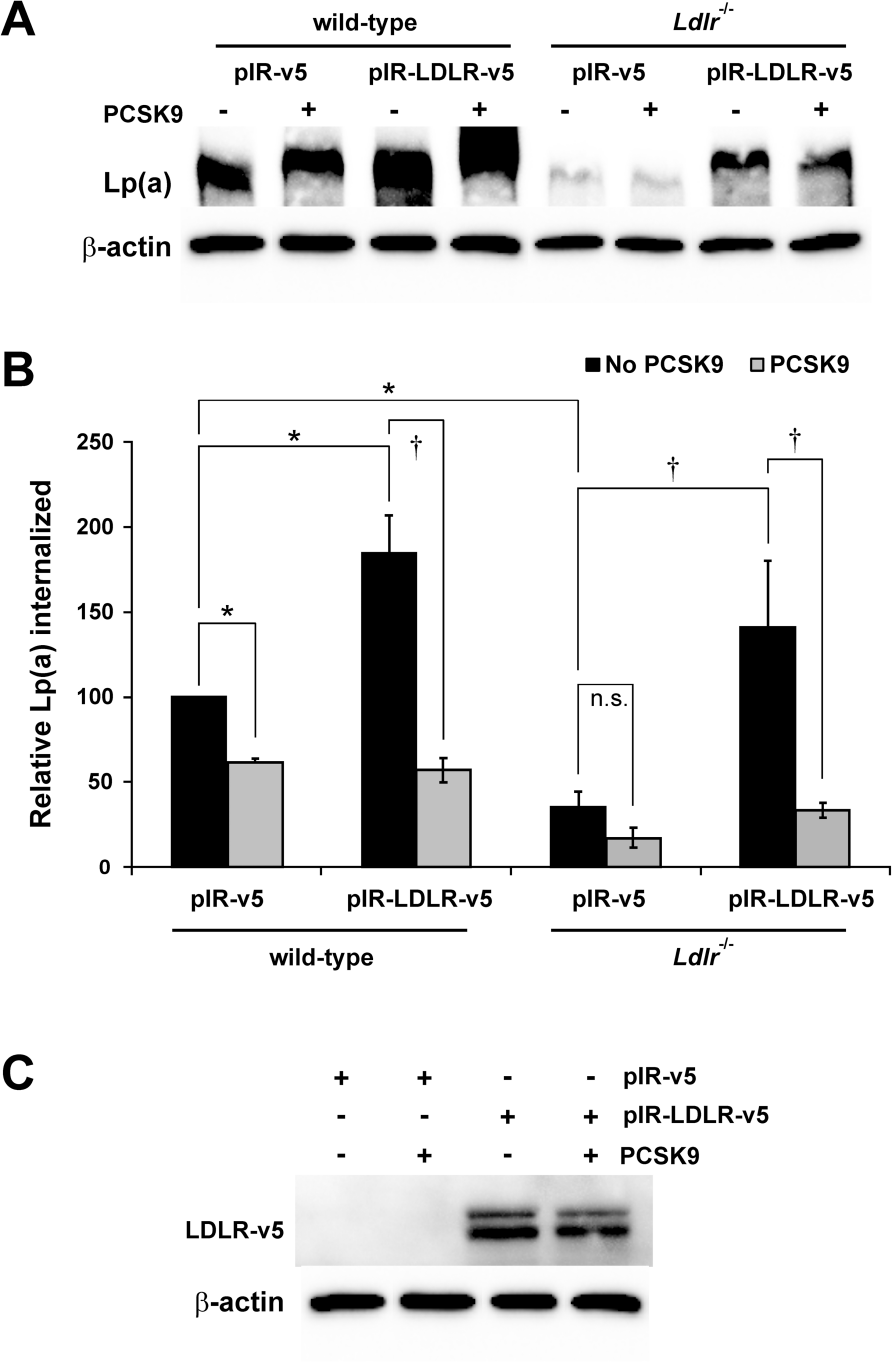
Internalization of Lp(a) by primary mouse hepatocytes. Hepatocytes were isolated from wild-type (left) and *Ldlr*^-/-^ (right) mice. Where indicated, cells were transiently transfected with an expression vector encoding human LDLR (pIR-LDLR-v5) or the corresponding empty expression vector (pIR-v5). Cells were incubated with 10 μg/mL Lp(a) in the absence or presence of 20 μg/mL PCSK9 for 4 hours. Cells were extensively washed to remove any bound Lp(a) and lysed to determine the relative amount of internalized Lp(a) compared to β-actin using western blot analysis. (A) Representative blots are shown. (B) graphical representation of the results, representing the means ± s.e.m. of at least 3 independent experiments. *: p < 0.05 versus control by one-sample t-test; †: p < 0.05 for indicated pairwise comparison by Student’s t-test; n.s.: not significant. (C) Representative western blot (from wild-type hepatocytes) showing overexpression of LDLR in the presence or absence of added PCSK9 using an anti-v5 antibody.

We then turned to primary mouse hepatocytes, which are more readily obtained in higher amounts and which offer the possibility of examining the effect of gene disruption on Lp(a) catabolism. We compared Lp(a) internalization in hepatocytes isolated from wild-type and *Ldlr*^-/-^ animals. Lp(a) internalization was significantly diminished in hepatocytes isolated from *Ldlr*^-/-^ mice and in wild-type hepatocytes treated with PCSK9 (Fig 3). When human LDLR was overexpressed, there was enhanced Lp(a) internalization in both wild-type and *Ldlr*^-/-^ hepatocytes (Fig 3). A significant decrease in Lp(a) internalization by the wild-type or *Ldlr*^-/-^ hepatocytes overexpressing human LDLR was observed in the presence of PCSK9 (Fig 3).

### Effect of PCSK9 mutations on Lp(a) internalization

**Fig 4:**
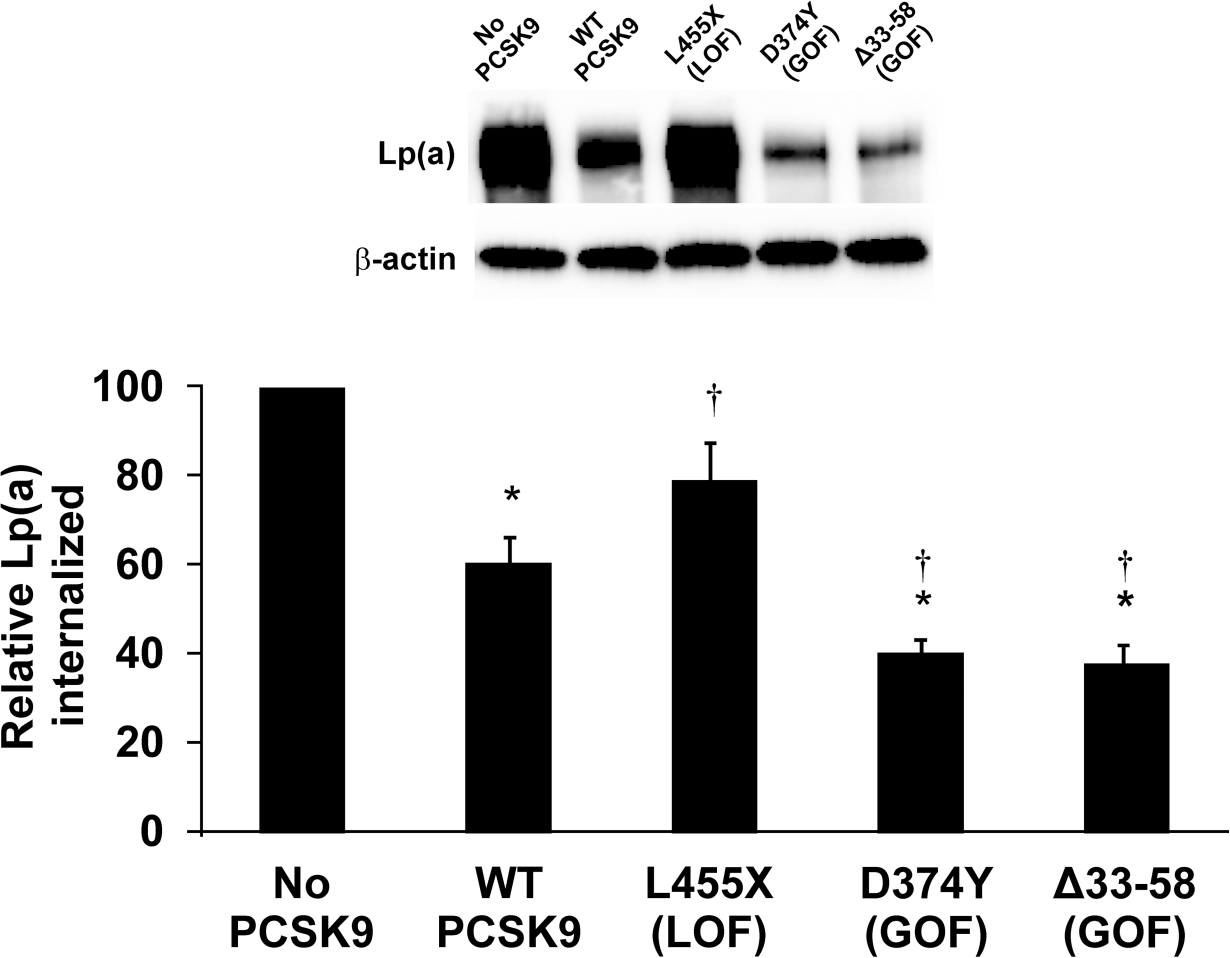
Effect of GOF and LOF variants of PCSK9 on Lp(a) internalization. HepG2 cells were treated with either no PCSK9, or 10 μg/mL purified PCSK9 variants. Cells were incubated with 10 μg/mL purified human Lp(a) for 4 hours. Cells were extensively washed to remove any bound Lp(a) and lysed to determine the relative amount of internalized Lp(a) compared to β-actin using western blot analysis. Representative blots are shown. The data represent the means ± s.e.m. of at least 4 independent experiments. *: *p* < 0.05 vs absence of PCSK9 by one-sample t-test; †: *p* < 0.05 versus wild-type (WT) PCSK9 by Student’s t-test.

Both gain-of-function (GOF) and loss-of-function (LOF) mutations in PCSK9 have been described, both present naturally in the human population and created by recombinant DNA techniques [43, 46, 47]. Expression plasmids encoding both LOF and GOF mutations were transfected into HepG2 cells and Lp(a) internalization measured. The D374Y mutation is a GOF that has been described in the human population and which binds to LDLR with a greater affinity, hence resulting in a decrease in LDLR receptor number on the hepatocyte surface [48]. Accordingly, this variant was more effective than wild-type PSCK9 at inhibiting Lp(a) internalization (Fig 4). We also tested the Δ33–58 variant, in which a segment of the prodomain that is inhibitory to the LDLR-reducing activity of PCSK9 has been removed, resulting in a more active protein (GOF mutation) [43]. As for the D374Y GOF variant, the Δ33–58 variant had enhanced ability to inhibit Lp(a) internalization (Fig 4). The L455X truncation mutant eliminates the carboxyl-terminal Cys-His-rich domain (CHRD), a domain critical for targeting LDLR, and thus acts as an LOF mutation [47]. This variant did not significantly decrease Lp(a) internalization relative to the control condition where no exogenous PCSK9 was added (Fig 4).

### Apo(a) isoform size-dependence of effect of PCSK9 on Lp(a) internalization

**Fig 5:**
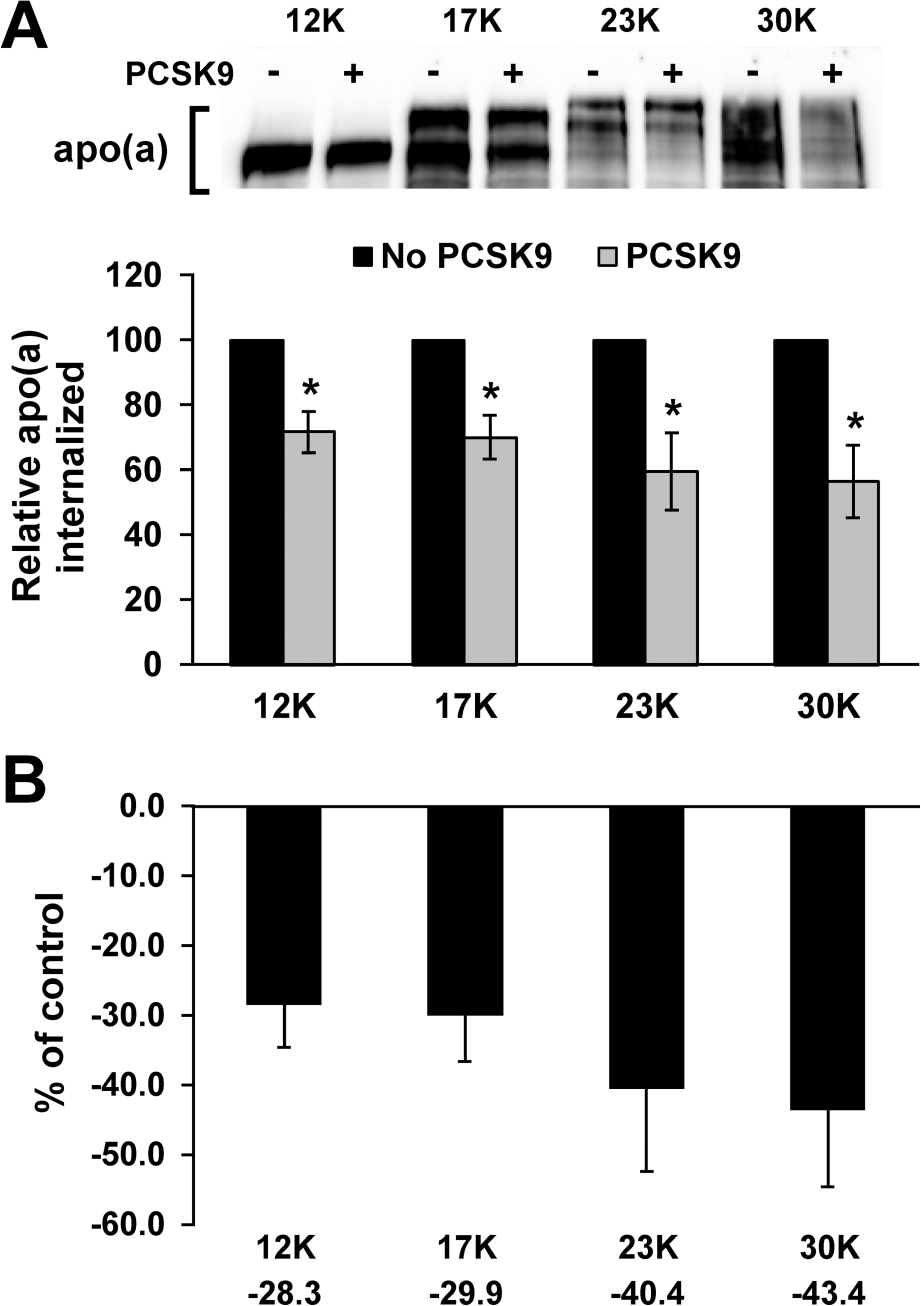
Effect of apo(a) isoform size on the ability of PCSK9 to regulate internalization. (A) HepG2 cells were treated with the indicated recombinant apo(a) variants (200 nM) in the presence or absence of 10 μg/mL purified PCSK9 for 4 hours. Cells were extensively washed to remove any bound apo(a) and lysed to determine the relative amount of internalized apo(a) compared to β-actin using western blot analysis. The internalization values in the presence of PCSK9 are expressed relative to the values obtained for that particular isoform in the absence of PCSK9. Representative blots are shown. The data represent the means ± s.e.m. of at least 7 independent experiments. *: *p* < 0.05 vs absence of PCSK9 by Student’s t-test. (B) The percent decrease in apo(a) internalization evoked by PCSK9 was calculated from the data in (A) and is plotted for each apo(a) isoform. No significant differences were observed (by one-way ANOVA).

There is a general inverse relationship between apo(a) isoform size and plasma Lp(a) concentrations. Although this has been demonstrated to be a consequence of differences in apo(a) synthesis and secretion, rather than catabolism [9], it remains possible that clearance through the LDLR might be affected by apo(a) isoform size. We utilized purified r-apo(a) variants ranging in size from 12 KIV repeats (12K) to 30, a range that represents > 90% of the isoforms observed in the human population [7]. We found that internalization of all isoforms was diminished by the addition of PCSK9 (Fig 5). Although there appeared to be a trend for a greater effect of PCSK9 with larger Lp(a) isoforms, none of the mean decreases for a particular isoform were significantly different from those for any other isoform (Fig 5B).

### Ability of LDLR-related receptors to internalize Lp(a): Role of PCSK9

**Fig 6:**
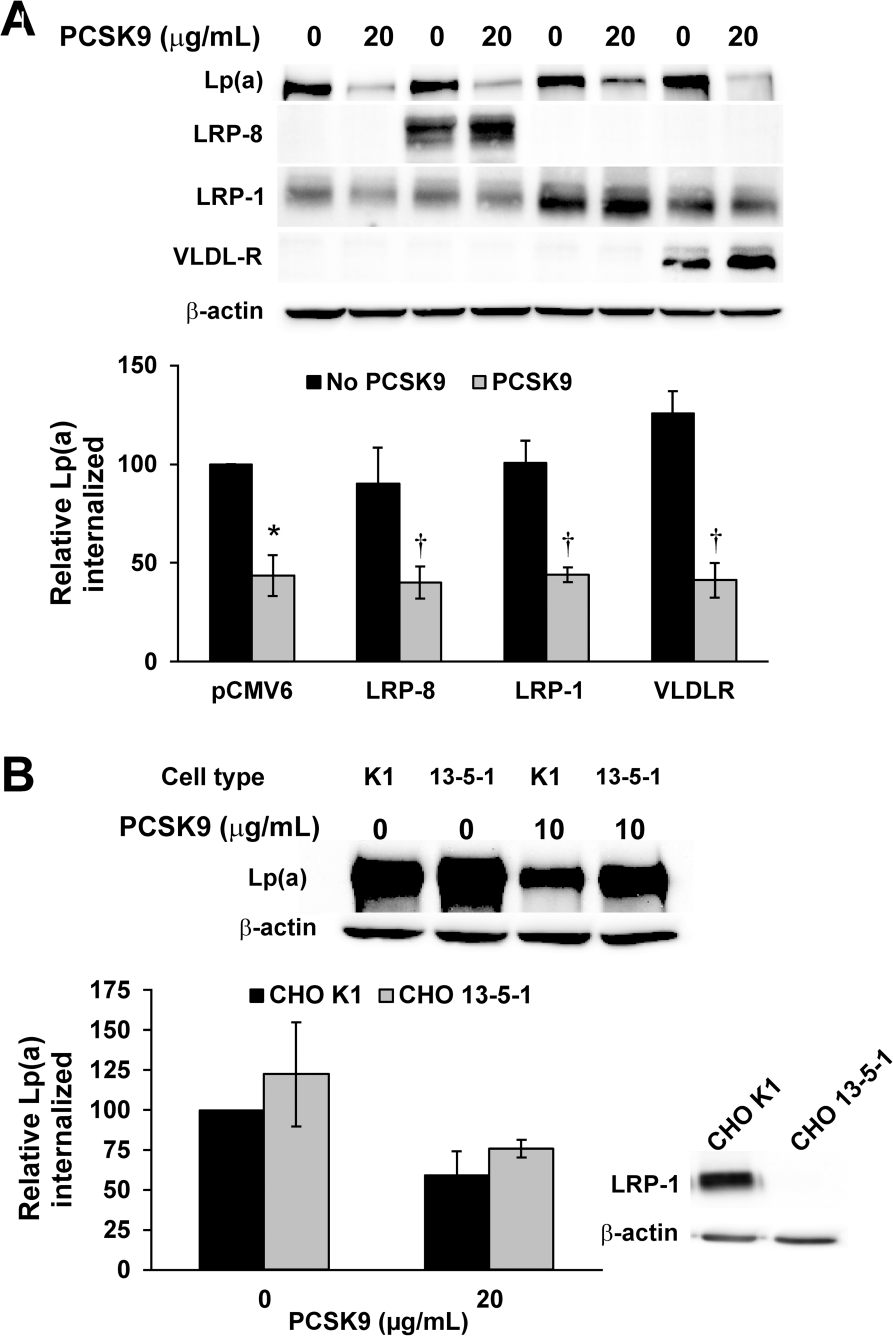
Role of LDLR-related receptors in Lp(a) internalization. (A) HepG2 cells were transiently transfected with the indicated expression vectors or the empty parental pCMV6 vector. Cells were incubated with 10 μg/mL purified human Lp(a) for 4 hours. Cells were extensively washed to remove any bound Lp(a) and lysed to determine the relative amount of internalized Lp(a) compared to β-actin using western blot analysis. Representative western blots for Lp(a), β-actin, and the respective ectopically-expressed receptors are shown. (B) Lp(a) internalization assays were performed as in Panel A, except in the LRP-1-expressing CHO cell line K1 or the LRP-deficient CHO cell line 13-5-1. Also shown is a western blot confirming the absence of LRP-1 in the 13-5-1 cell line. The data represent the means ± s.e.m. of at least 3 independent experiments. *: *p* < 0.05 vs absence of PCSK9 by one-sample t-test; †: *p* < 0.05 versus absence of PCSK9 by Student’s t-test.

Several reports have indicated that other receptors from the LDLR family can be regulated by PCSK9, namely LRP-1 [40], LRP-8 (also known as apoER2) [49], and VLDLR [49]; LRP-1 and VLDLR have also been proposed as candidate receptors for Lp(a) [18, 19]. We therefore transfected expression vectors encoding each of these receptors in HepG2 cells to assess if they may participate in Lp(a) internalization. LRP-8 and VLDLR are not endogenously expressed in HepG2 cells, while LRP-1 is, as assessed by western blot analysis (Fig 6A). In no case did overexpression of any of these receptors significantly enhance Lp(a) internalization (Fig 6A). These data are in stark contrast to what is observed when LDLR is overexpressed in HepG2 cells: we observed close to a 10-fold increase in Lp(a) internalization in this circumstance [37]. We also noted that overexpression of LRP-1, LRP-8, or VLDLR did not affect the decrease in Lp(a) internalization caused by the addition of purified PCSK9 (Fig 6A).

Transfection with expression vectors for the respective receptors results in a 1300-fold increase in VLDLR mRNA, a 1700-fold increase in LRP-8 mRNA, but only a 1.5-fold increase in LRP-1 mRNA. Therefore, to verify the apparent lack of a role for LRP-1, we utilized CHO cell lines either expressing (K1) or lacking (13-5-1) LRP-1. There was no significant difference in Lp(a) internalization between the two cell types, either in the presence or absence of added PCSK9 (Fig 6B). PCSK9 appeared to decrease Lp(a) internalization in K1 cells; PCSK9 also appeared to decrease Lp(a) internalization in 13-5-1 cells, although the decreases did not reach significance.

## Discussion

The discovery that antibody inhibitors of PCSK9 lower plasma Lp(a) concentrations triggered excitement in the Lp(a) field, but also presented a paradox: given the apparent lack of a role for the LDLR in Lp(a) catabolism, what could be the mechanism behind the effect of these therapies? Our own studies, and those of others, using *in vitro* internalization assays with HepG2 cells indicated that Lp(a) was a *bona fide* ligand for the LDLR, and that PCSK9 inhibited internalization of Lp(a) through this route by decreasing LDLR abundance [34, 37]. We argued that, under normal circumstances when LDL is considerably more abundant than Lp(a) in plasma, the LDLR is indeed not a major route of Lp(a) catabolism. However, in the setting of PCSK9 inhibitors, a situation is set up where LDLR levels in the liver are supraphysiological and LDL plasma concentrations are markedly reduced; in this circumstance, LDLR could play a greater role in Lp(a) clearance. This concept has been supported by data that the extent of the decrease in Lp(a) levels elicited by PCSK9 inhibitors is correlated with the decrease in LDL levels, although one study did not detect such a correlation (see above). A recently study using *in vivo* tracer kinetic studies suggested that the PCSK9 inhibitor alirocumab increased the fractional catabolic rate of apo(a), but not its production rate [50]. On the other hand, *in vivo* tracer kinetic studies centered on the effect of extended release niacin (ERN) on Lp(a) metabolism failed to show a connection between changes in plasma concentrations of PCSK9 and Lp(a) catabolic rate; this suggests against a role for LDLR in Lp(a) clearance [51, 52]. A recent study in primary human hepatocytes reported that PCSK9 increases Lp(a) biosynthesis, an effect that is blocked by alirocumab[53]. Finally, a recent study suggests that PCSK9 can bind to Lp(a) as it does to LDL [54], an interaction we could not detect using direct binding assays [37]. It has been suggested that Lp(a), PCSK9, and the PCSK9 therapeutic antibody can form a ternary complex that can then bind the Fc receptor and be cleared by the reticuloendothelial system [55]. However, Phase I studies of PCSK9 inhibition using an RNAi therapeutic showed a similar decrease in plasma Lp(a) concentrations [56], thereby casting doubt on this proposed mechanism.

The question of why Lp(a) is resistant to lowering by statins if the LDLR can function as a route of catabolism for Lp(a) remains an interesting paradox. It should be noted that statins, in addition to increasing LDLR number, also increase hepatic PCSK9 production which can counteract the statin effect [57]. Moreover, since LDL is always more abundant than Lp(a), there is a requirement for very high LDLR numbers and reduced levels of competing LDL for LDLR-mediated internalization of Lp(a) to be observable. Hence, PCSK9 inhibitors, either in the presence or absence of statins, elicit a significant contribution of LDLR to Lp(a) clearance that statins alone cannot.

In this context, the goal of the present study was to obtain independent evidence for a role for the LDLR in Lp(a) internalization, and to explore other receptors through which PCSK9 could act to promote Lp(a) clearance. We found, in three different hepatocyte culture models (Huh7 hepatoma cells, primary human hepatocytes, and primary mouse hepatocytes), that PCSK9 promotes Lp(a) internalization. In the primary human hepatocytes, we were not able to directly examine Lp(a) internalization because we found that Lp(a) adhered very tightly to the collagen matrix that is required for growth of these cells. Even if we included ε-ACA in the incubation, there was no decrement in the very intense apo(a) band that we observed in the lysates by western blot analysis. Normally, ε-ACA drastically impairs apo(a)/Lp(a) internalization, as observed in Fig 2. In this experiment using the primary human hepatocytes, ε-ACA inhibits apo(a) internalization in two ways: (i) by interfering with the lysine-dependent interaction between apo(a) and cell-surface plasminogen receptors (likely the major component [13]; and (ii) by preventing non-covalent interactions between apo(a) and apoB-100 that allow apo(a) catabolism through the LDLR in a “piggy-backing” mechanism. Yet, no inhibition of Lp(a) internalization was observed (S2 Fig). In a key experiment, when Lp(a) was incubated with the collagen matrix alone followed by the same washing protocol culminating in the addition of lysis buffer, a similar intensity of apo(a) was observed as was the case when the primary hepatocytes were present (S3 Fig). It is unclear if this apparent non-specific binding might be behind the apparent lack of effect of PCSK9 on Lp(a) internalization by primary human hepatocytes that has been reported by others [53].

Our experiments with mouse primary hepatocytes suggest that the absence of the LDLR led to a diminution of the extent of Lp(a) internalization and the disappearance of the ability of PCSK9 to downregulate Lp(a) internalization (Fig 3). However, some of the apparent differences did reach statistical significance in ANOVA analysis, likely because of the high degree of variability in the data when LDLR was overexpressed. In corresponding clearance studies performed in mice of the same genotype as that which yielded our primary hepatocytes, absence of the LDLR did not have a significant effect on Lp(a) catabolism [22]. In agreement with our findings, however, overexpression of human LDLR in mice did promote Lp(a) clearance [25]. Once again, the relative concentrations of Lp(a) versus other LDLR ligands may be relevant, as the amount of Lp(a) used in the experiments with LDLR-deficient mice was not reported [22]. It is also possible that, *in vivo*, absence of LDLR has less of an effect on Lp(a) catabolism than overexpression of LDLR, since this receptor only plays minor role, if any, under basal conditions.

Another line of evidence in support of our model for the effect of PCSK9 inhibitors on Lp(a) levels comes from our study of certain PCSK9 mutants. As in our previous study, we found that the GOF mutant D374Y, which binds LDLR more tightly, correspondingly had a greater effect than wild-type PCSK9 in inhibiting Lp(a) internalization in our model (Fig 4) [37]. Similarly, the Δ33–58 GOF mutation was more effective than the wild-type PCSK9, while the L455X LOF mutation resulted in a reduced ability to promote Lp(a) internalization (Fig 4). Since the phenotype of each of these mutants depends on a productive interaction (or lack thereof) with LDLR, these findings demonstrate again that the effects of PCSK9 on Lp(a) internalization are dependent on LDLR.

Although it has been reported that Lp(a) isoform size has no effect on the catabolic rate of Lp(a) [9], it is not yet known if PCSK9 can differentially regulate the internalization of differently-sized Lp(a) isoforms. Our findings (Fig 5B) show that there is no difference between the fractional decrease in apo(a) internalization attributable to PCSK9. In agreement with previous findings [9], no significant difference was observed in the internalization of smaller versus larger isoforms (S4 Fig). Clinical trial data with PCSK9 inhibitory antibodies show that the percentage decrease in Lp(a) is greater for subjects with lower Lp(a) but the absolute decrease is greater for subjects with high Lp(a) [30, 31]. Although the frequency of small isoform sizes would be greater in subjects with high Lp(a), in general Lp(a) isoform size has not been directly measured in study patients, and as such the relationship between Lp(a) isoform size and the effect of PCSK9 cannot be assessed in these studies.

Several studies have suggested that PCSK9 can regulate the abundance of several receptors in the LDLR family besides LDLR itself [40, 49]. These include LRP-8, LRP-1, and VLDLR, the last two of which have also been implicated as Lp(a) receptors [18, 19]. However, overexpression of each of these receptors in HepG2 cells failed to promote Lp(a) internalization (Fig 6). Unsurprisingly, the ability of PCSK9 to decrease Lp(a) internalization was unaffected by overexpression of these receptors. These finding are in keeping with our previous results using anti-LDLR inhibitory antibodies that demonstrated that the entirety of the PCSK9-sensitive component of Lp(a) internalization in HepG2 cells was accounted for by its effects on the LDLR [37].

Our results underscore the ability of the LDLR to act as a clearance receptor for Lp(a) in hepatocytes and are consistent with our hypothesis that anti-PCSK9 drugs promote Lp(a) clearance by increasing LDLR abundance in the liver. However, they do not exclude that other mechanisms may account for the observed effects of PCSK9 inhibitors. For instance, it remains possible that inhibition of PCSK9 decreases Lp(a) biosynthesis, such as by inhibiting secretion of apoB-containing lipoproteins [44] or limiting the availability of apoB-containing lipoproteins for assembly with apo(a) to form Lp(a) particles. *In vitro* cell model, animal model, and human metabolic experiments will be necessarily to definitively unravel all of these possibilities.

## Supporting information

S1 Fig Quantitative analysis of Lp(a) internalization. Western blot analysis was performed on a series of samples representing different quantities of purified Lp(a) as well as a lysate of HepG2 cells exposed to 10 μg/mL Lp(a) for 4 hours. The grey lines represent the interpolated quantity of Lp(a) from the density of the Lp(a) lysate band.
(PDF)

S2 Fig Lp(a) internalization by primary hepatocytes. Hepatocytes were plated on a collagen matrix and then incubated with 200 nM Lp(a) in the absence or presence of the indicated concentrations of PCSK9 or 200 mM ε-ACA for 4 hours. Cells were extensively washed to remove any bound Lp(a) and then lysed. Western blot analysis was used to determine the relative amount of internalized Lp(a); β-actin was used as an internal standard. A representative blot is shown. Note the comparative inability of ε-ACA to compete for Lp(a) internalization (compare to Fig 2).
(PDF)

S3 Fig Non-specific Lp(a) binding to collagen surfaces. A collagen matrix was prepared but seeding of hepatocytes was omitted. The matrix was then incubated with 200 nM Lp(a) for 4 hours. Wells were subjected to several different wash conditions as described below, at either 4°C or 37°C, and then lysed. Western blot analysis was used to determine the relative amount of internalized Lp(a). A representative blot is shown. Lane 1: 3× wash with PBS. Lane 2: 10× wash with PBS containing 0.5 M NaCl. Lane 3: 10× wash with PBS containing 0.5 M NaCl and 1% BSA. Lane 4: 10× wash with PBS containing 0.5 M NaCl, 1% BSA, and 200 mM ε-ACA. Lane 5: 10× wash with PBS containing 0.5 M NaCl, 1% BSA, and 200 mM ε-ACA, followed by an acid wash with 0.2 M acetic acid pH 2.5 containing 0.5 M NaCl. Lane 6: 3× with PBS, 0.8% BSA, 2× with PBS containing 10 μg/ml heparin for 10 min, 1× with PBS, BSA, 0.2 M ε-ACA for 5 min; 2× with 0.2 M acetic acid, pH 2.5, containing 0.5 M NaCl for 10 min, 1× with 0.5 M HEPES, pH 7.5, 100 mM NaCl for 10 min, 1× with PBS (Lane 6 represents the normal washing conditions we employed elsewhere in this study). Note that progressively more extensive and harsh washing conditions appeared to actually promote binding to the collagen surfaces.
(PDF)

S4 Fig Effect of isoform size on apo(a) internalization. HepG2 cells were treated with the indicated recombinant apo(a) variants (200 nM) for 4 hours. Cells were extensively washed to remove any bound apo(a) and lysed to determine the relative amount of internalized apo(a) compared to β-actin using western blot analysis. The internalization values are expressed relative to that of 12K. The data represent the means ± s.e.m. of at least 7 independent experiments. No significant differences compared to 12K were observed (by one-sample t-test).
(PDF)
