# Supplementary material for: Roles of the low density lipoprotein receptor and related receptors in inhibition of lipoprotein(a) internalization by proprotein convertase subtilisin/kexin type 9
Source: PLoS One. 2017 Jul 27;12(7):e0180869. doi: 10.1371/journal.pone.0180869 (PMC5531514; doi:10.1371/journal.pone.0180869)
Supplement: S1 Fig — Western blot analysis was performed on a series of samples representing different quantities of purified Lp(a) as well as a lysate of HepG2 cells exposed to 10 μg/mL Lp(a) for 4 hours. The grey lines represent the interpolated quantity of Lp(a) from the density of the Lp(a) lysate band. (PDF) [file pone.0180869.s001.pdf]

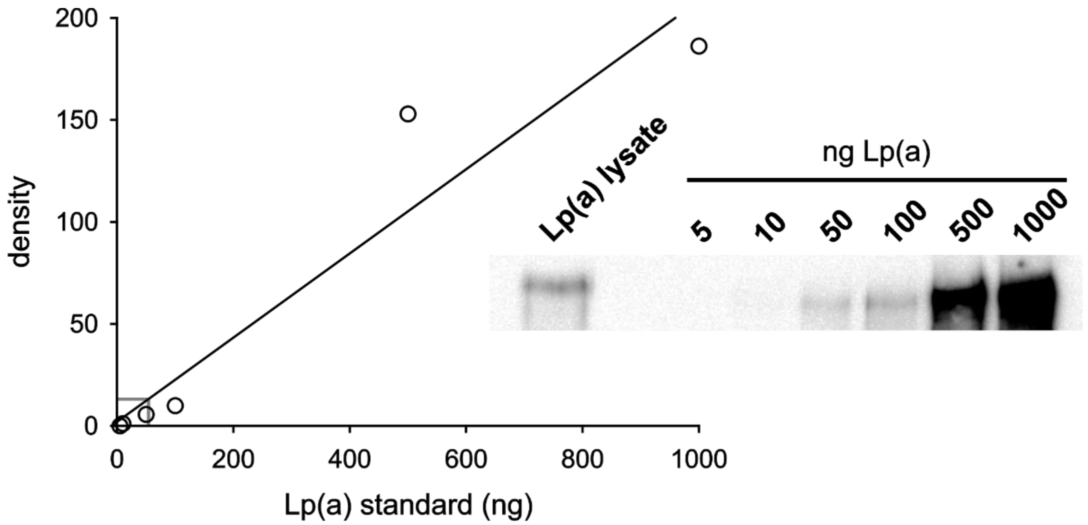

S1 Fig. Quantitative analysis of Lp(a) internalization. Western blot analysis was performed on a series of samples representing different quantities of purified Lp(a) as well as a lysate of HepG2 cells exposed to 10 µg/mL Lp(a) for 4 hours. The grey lines represent the interpolated quantity of Lp(a) from the density of the Lp(a) lysate band.
