# Supplementary material for: Roles of the low density lipoprotein receptor and related receptors in inhibition of lipoprotein(a) internalization by proprotein convertase subtilisin/kexin type 9
Source: PLoS One. 2017 Jul 27;12(7):e0180869. doi: 10.1371/journal.pone.0180869 (PMC5531514; doi:10.1371/journal.pone.0180869)
Supplement: S2 Fig — Hepatocytes were plated on a collagen matrix and then incubated with 200 nM Lp(a) in the absence or presence of the indicated concentrations of PCSK9 or 200 mM ε-ACA for 4 hours. Cells were extensively washed to remove any bound Lp(a) and then lysed. Western blot analysis was used to determine the relative amount of internalized Lp(a); β-actin was used as an internal standard. A representative blot is shown. Note the comparative inability of ε-ACA to compete for Lp(a) internalization (compare to Fig 2). (PDF) [file pone.0180869.s002.pdf]

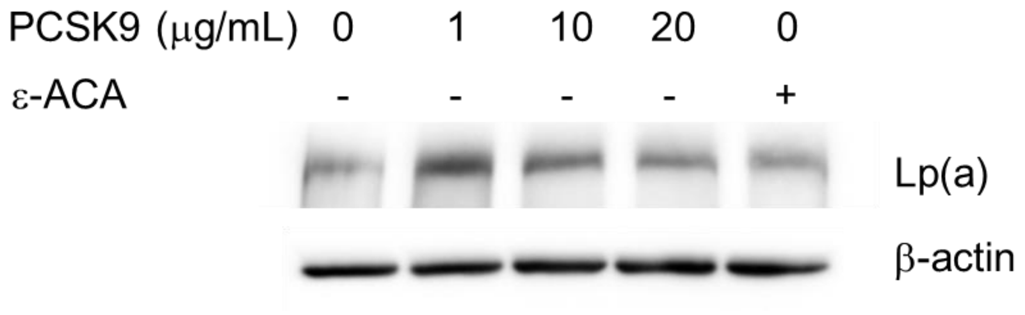

S2 Fig. Lp(a) internalization by primary hepatocytes. Hepatocytes were plated on a collagen matrix and then incubated with 200 nM Lp(a) in the absence or presence of the indicated concentrations of PCSK9 or 200 mM ε-ACA for 4 hours. Cells were extensively washed to remove any bound Lp(a) and then lysed. Western blot analysis was used to determine the relative amount of internalized Lp(a); β-actin was used as an internal standard. A representative blot is shown. Note the comparative inability of ε-ACA to compete for Lp(a) internalization (compare to Fig. 2).
