# Supplementary material for: Roles of the low density lipoprotein receptor and related receptors in inhibition of lipoprotein(a) internalization by proprotein convertase subtilisin/kexin type 9
Source: PLoS One. 2017 Jul 27;12(7):e0180869. doi: 10.1371/journal.pone.0180869 (PMC5531514; doi:10.1371/journal.pone.0180869)
Supplement: S3 Fig — A collagen matrix was prepared but seeding of hepatocytes was omitted. The matrix was then incubated with 200 nM Lp(a) for 4 hours. Wells were subjected to several different wash conditions as described below, at either 4°C or 37°C, and then lysed. Western blot analysis was used to determine the relative amount of internalized Lp(a). A representative blot is shown. Lane 1: 3× wash with PBS. Lane 2: 10× wash with PBS containing 0.5 M NaCl. Lane 3: 10× wash with PBS containing 0.5 M NaCl and 1% BSA. Lane 4: 10× wash with PBS containing 0.5 M NaCl, 1% BSA, and 200 mM ε-ACA. Lane 5: 10× wash with PBS containing 0.5 M NaCl, 1% BSA, and 200 mM ε-ACA, followed by an acid wash with 0.2 M acetic acid pH 2.5 containing 0.5 M NaCl. Lane 6: 3× with PBS, 0.8% BSA, 2× with PBS containing 10 μg/ml heparin for 10 min, 1× with PBS, BSA, 0.2 M ε-ACA for 5 min; 2× with 0.2 M acetic acid, pH 2.5, containing 0.5 M NaCl for 10 min, 1× with 0.5 M HEPES, pH 7.5, 100 mM NaCl for 10 min, 1× with PBS (Lane 6 represents the normal washing conditions we employed elsewhere in this study). Note that progressively more extensive and harsh washing conditions appeared to actually promote binding to the collagen surfaces. (PDF) [file pone.0180869.s003.pdf]

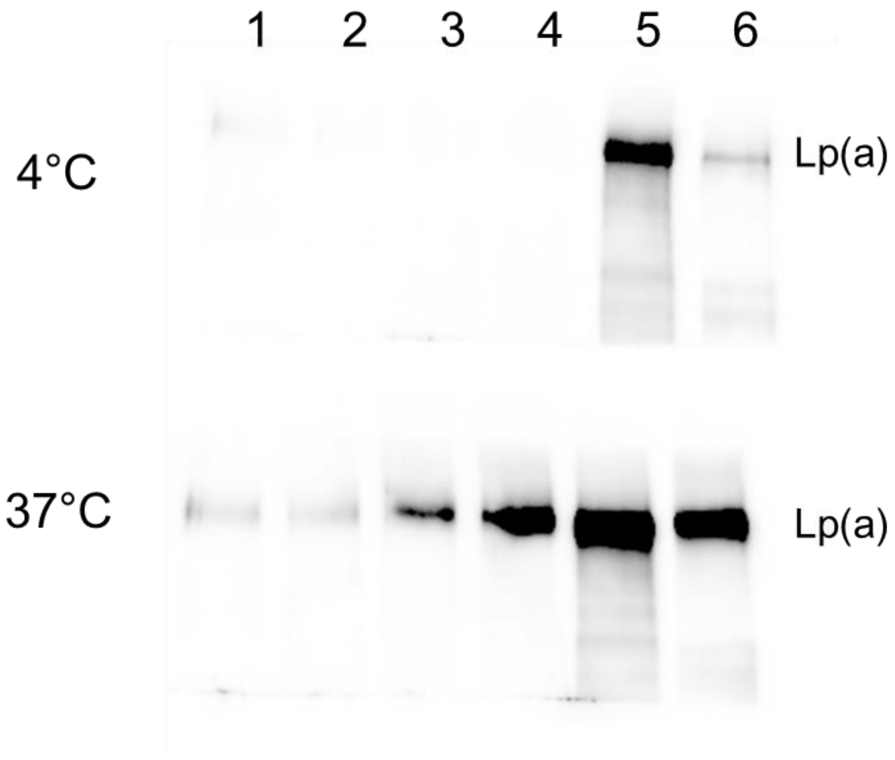

S3 Fig. Non-specific Lp(a) binding to collagen surfaces. A collagen matrix was prepared but seeding of hepatocytes was omitted. The matrix was then incubated with 200 nM Lp(a) for 4 hours. Wells were subjected to several different wash conditions as described below, at either 4°C or 37°C, and then lysed. Western blot analysis was used to determine the relative amount of internalized Lp(a). A representative blot is shown. Lane 1: 3× wash with PBS. Lane 2: 10× wash with PBS containing 0.5 M NaCl. Lane 3: 10× wash with PBS containing 0.5 M NaCl and 1% BSA. Lane 4: 10× wash with PBS containing 0.5 M NaCl, 1% BSA, and 200 mM ε-ACA. Lane 5: 10× wash with PBS containing 0.5 M NaCl, 1% BSA, and 200 mM ε-ACA, followed by an acid wash with 0.2 M acetic acid pH 2.5 containing 0.5 M NaCl. Lane 6: 3× with PBS, 0.8% BSA, 2× with PBS containing 10 μg/ml heparin for 10 min, 1× with PBS, BSA, 0.2 M ε-ACA for 5 min; 2× with 0.2 M acetic acid, pH 2.5, containing 0.5 M NaCl for 10 min, 1× with 0.5 M HEPES, pH 7.5, 100 mM NaCl for 10 min, 1× with PBS (Lane 6 represents the normal washing conditions we employed elsewhere in this study). Note that progressively more extensive and harsh washing conditions appeared to actually promote binding to the collagen surfaces.
