# Supplementary material for: Roles of the low density lipoprotein receptor and related receptors in inhibition of lipoprotein(a) internalization by proprotein convertase subtilisin/kexin type 9
Source: PLoS One. 2017 Jul 27;12(7):e0180869. doi: 10.1371/journal.pone.0180869 (PMC5531514; doi:10.1371/journal.pone.0180869)
Supplement: S4 Fig — HepG2 cells were treated with the indicated recombinant apo(a) variants (200 nM) for 4 hours. Cells were extensively washed to remove any bound apo(a) and lysed to determine the relative amount of internalized apo(a) compared to β-actin using western blot analysis. The internalization values are expressed relative to that of 12K. The data represent the means ± s.e.m. of at least 7 independent experiments. No significant differences compared to 12K were observed (by one-sample t-test). (PDF) [file pone.0180869.s004.pdf]

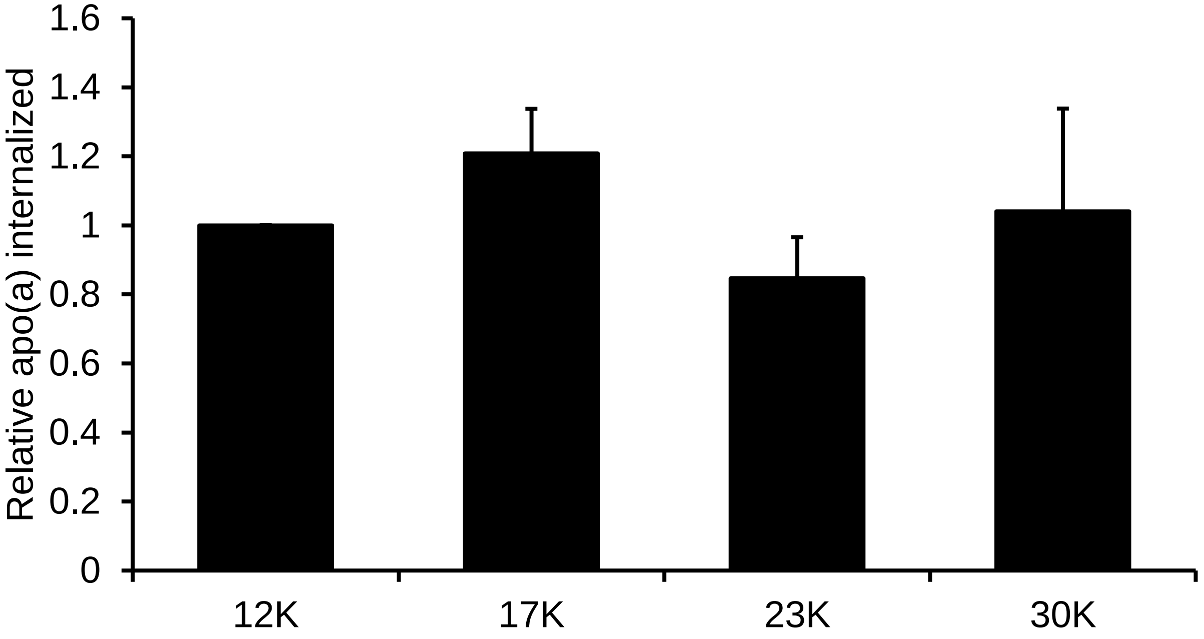

S4 Fig. Effect of isoform size on apo(a) internalization. HepG2 cells were treated with the indicated recombinant apo(a) variants (200 nM) for 4 hours. Cells were extensively washed to remove any bound apo(a) and lysed to determine the relative amount of internalized apo(a) compared to β-actin using western blot analysis. The internalization values are expressed relative to that of 12K. The data represent the means ± s.e.m. of at least 7 independent experiments. No significant differences compared to 12K were observed (by one-sample t-test).
